# Fibrinogen/albumin ratio as a promising predictor of platinum response and survival in ovarian clear cell carcinoma

**DOI:** 10.1186/s12885-022-09204-0

**Published:** 2022-01-21

**Authors:** Wei Chen, Boer Shan, Shuling Zhou, Huijuan Yang, Shuang Ye

**Affiliations:** 1 Department of Gynecologic Oncology, Fudan University Shanghai Cancer Center, Shanghai, China; 2 Department of Obstetrics and Gynecology, Minhang Hospital, Fudan University, the Central Hospital of Minhang District, Shanghai, China; 3 Department of Oncology, Shanghai Medical College, Fudan University, Shanghai, 200032 China; 4 Department of Pathology, Fudan University Shanghai Cancer Center, Shanghai, China

**Keywords:** Ovarian neoplasms, Clear cell carcinoma, Fibrinogen/albumin ratio, Platinum resistance, Survival

## Abstract

**Background:**

This study aims to evaluate the role of the fibrinogen/albumin ratio (FAR) in predicting platinum resistance and survival outcomes of patients with ovarian clear cell carcinoma (OCCC).

**Methods:**

Coagulation function and D-dimer, serum albumin, CA125 and HE4 levels were measured before surgery in OCCC patients undergoing initial surgery in our institution. FAR was calculated as fibrinogen/albumin level. The correlation between these indicators and clinicopathological features, platinum response, and survival outcomes was further analyzed. The Kaplan-Meier method and multivariable Cox regression model were used to assess the effects of FAR on progression-free survival (PFS) and overall survival (OS).

**Results:**

Advanced stage patients accounted for 42.1% of the 114 participants. Optimal cytoreductive surgery was achieved in 105 patients, and the complete resection rate was 78.1%. FAR was associated with tumor stage, residual tumor and platinum response. A receiver operating characteristic curve for predicting platinum response showed that the optimal cutoff point of the FAR was 12%. The sensitivity was 73.3% and the specificity was 68.2%. In multivariate analysis, FAR ≥12% (HR = 4.963, *P* = 0.002) was an independent risk factor for platinum resistance. In addition, FAR and D-dimer proved to be independent negative factors for outcomes including both PFS and OS. The median follow-up time was 52 months. A high FAR (≥ 12%) showed a stronger correlation with poor OS and PFS in the subgroup analysis of advanced and completely resected patients.

**Conclusions:**

The FAR might be a potential preoperative biochemical marker for predicting treatment response and oncological outcomes in OCCC patients.

## Background

Ovarian cancer is one of the most lethal diseases among gynecological malignancies [1]. Seventy percent of ovarian cancers are epithelial, of which ovarian clear cell carcinoma (OCCC) is a specific type of epithelial ovarian cancer (EOC) with a higher incidence in East Asian women [2]. Tumor stage, residual tumor and chemotherapy response are recognized as prognostic factors for survival in ovarian cancer [3]. Although many patients with OCCC are diagnosed at an early stage, their prognosis is still worse due to disease aggressiveness and platinum resistance compared to their serous counterparts, which account for the highest proportion [4, 5]. In addition, OCCC patients who harbor the same stage or residual tumors also show obvious heterogeneous survival due to their unpredictable response to platinum [6]. The high cost of genetic markers limits their application in clinical practice. Therefore, a simple, economical and effective biomarker that accurately reflects the platinum response and survival outcome of the disease is needed.

Hypercoagulability is associated with malignancy and is more pronounced in patients with OCCC [7]. For example, patients with OCCC tend to show higher D-dimer than other patients with EOC [8]. In addition, fibrinogen, one of the important indicators of the coagulation function, is also an acute-phase protein, and its plasma level increases during the systemic inflammatory response [9]. It has attracted increasing attention because of its important role in the development and progression of inflammation and cancer [10–12]. Many studies on malignant tumors have shown that elevated plasma fibrinogen levels before surgery are significantly associated with treatment failure or adverse outcomes in patients [11]. Furthermore, the fibrinogen/albumin ratio (FAR) is considered to be an important biomarker reflecting the systemic inflammatory state and nutritional state. It has been reported that FAR is closely related to the prognosis of various cancers [9, 13–17]. However, few studies have explored the value of the FAR in OCCC.

Therefore, we tried to evaluate the role of the FAR as a predictive and prognostic biochemical marker in OCCC in a well-annotated cohort involving 114 patients treated in our institution.

## Materials and methods

### Patients

This retrospective study was approved by the Ethics Committee of Fudan University Shanghai Cancer Center, and the written informed consent requirement was waived due to its retrospective design. We searched our electronic medical record systems for all OCCC patients who underwent their initial surgery at our institution between 2007 and 2018. The inclusion criteria were as follows: 1) pathological diagnosis of OCCC; 2) no preoperative neoadjuvant chemotherapy; 3) no other malignant tumor; and 4) no signs of venous thromboembolism, including deep vein thrombosis and pulmonary embolism, at first diagnosis.

The interval between preoperative blood tests and surgery is usually less than seven days. Coagulation function tests (including fibrinogen), plasma D-dimer, serum albumin, HE4 and CA125 in routine preoperative examinations were retrospectively extracted from medical records. FAR is defined as the fibrinogen/albumin ratio. Comprehensive staging surgery was performed for the early-stage patients (stage I + II), and debulking reduction was performed for the advanced patients (stage III + IV). Optimal cytoreductive surgery means that the size of the residual tumor is less than or equal to 1 cm. Complete resection means no visible tumor after cytoreduction. Both recurrence and progression after treatment are based on imaging evidence (CT or MRI). Platinum resistance was defined as those who completed standard platinum-based chemotherapy and progressed within six months after the last chemotherapy session. Progression-free survival (PFS) and overall survival (OS) were defined as the time interval between the date of the primary surgery to the date of first recurrence and death/last contact, respectively.

In our institution, patients with venous thromboembolism (VTE) at first diagnosis usually receive anticoagulation (low molecular weight heparin during the study period) and neoadjuvant chemotherapy. Therefore, those patients were not included in the study considering that VTE, anticoagulation treatment and neoadjuvant chemotherapy might all affect the serum coagulation panels to different extents. During the study period, ovarian cancer patients usually receive platinum-based chemotherapy (paclitaxel 175 mg/m2 and carboplatin AUC = 5) after surgery. Patients with advanced tumors are required to receive at least six cycles. For those with early-stage disease, the number of cycles ranged from four to six after being tailored to different individuals. Postoperative radiation and maintenance treatment were not recommended.

### Statistical analysis

Clinical variables were described by descriptive statistics. Median and range were used for continuous variables, and proportions were used for classified data. Receiver operating characteristic (ROC) curves was established, and the optimal cutoff threshold of the FAR for predicting platinum response, PFS and OS were determined by Youden’s index correction. The baseline features compared using the Mann-Whitney U test due to nonnormal distribution. Univariate and multivariate analyses were performed based on the log-rank test and Cox regression, respectively. Survival time was estimated by the Kaplan-Meier model. All reported *P* values were double-tailed, and *P* < 0.05 was considered statistically significant. Statistical analyses were performed with SPSS Version 24.0 (SPSS, Inc., Chicago, IL, USA). ROC curves and Kaplan–Meier curves were plotted using GraphPad Prism 6.0 (GraphPad Software, Inc., La Jolla, CA).

## Result

### Clinical characteristics of the study population and the association between FAR and platinum response

**Fig. 1:**
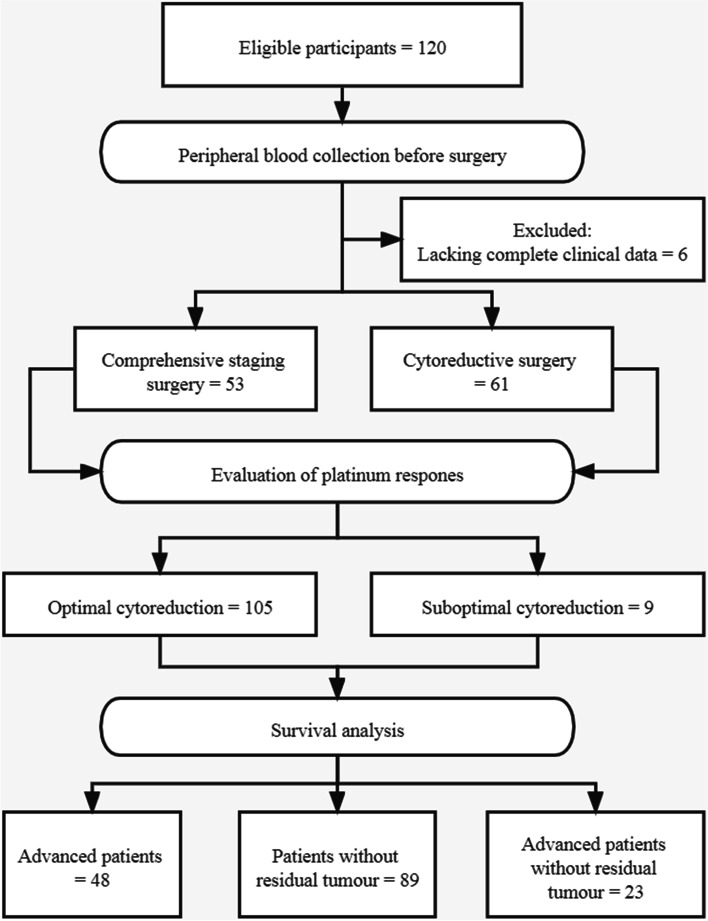
Flow chart illustrating the patient inclusion

A total of 120 OCCC patients met the eligibility criteria, and six patients were excluded for incomplete clinical data. Of these, 66 patients (57.9%) with early-stage disease (I + II) received staging surgery, and 48 patients (42.1%) with advanced-stage disease (III + IV) underwent debulking surgery. Optimal cytoreductive surgery was achieved in 105 patients. The flow chart of study participant inclusion and exclusion is shown in Fig. 1.

**Fig. 2:**
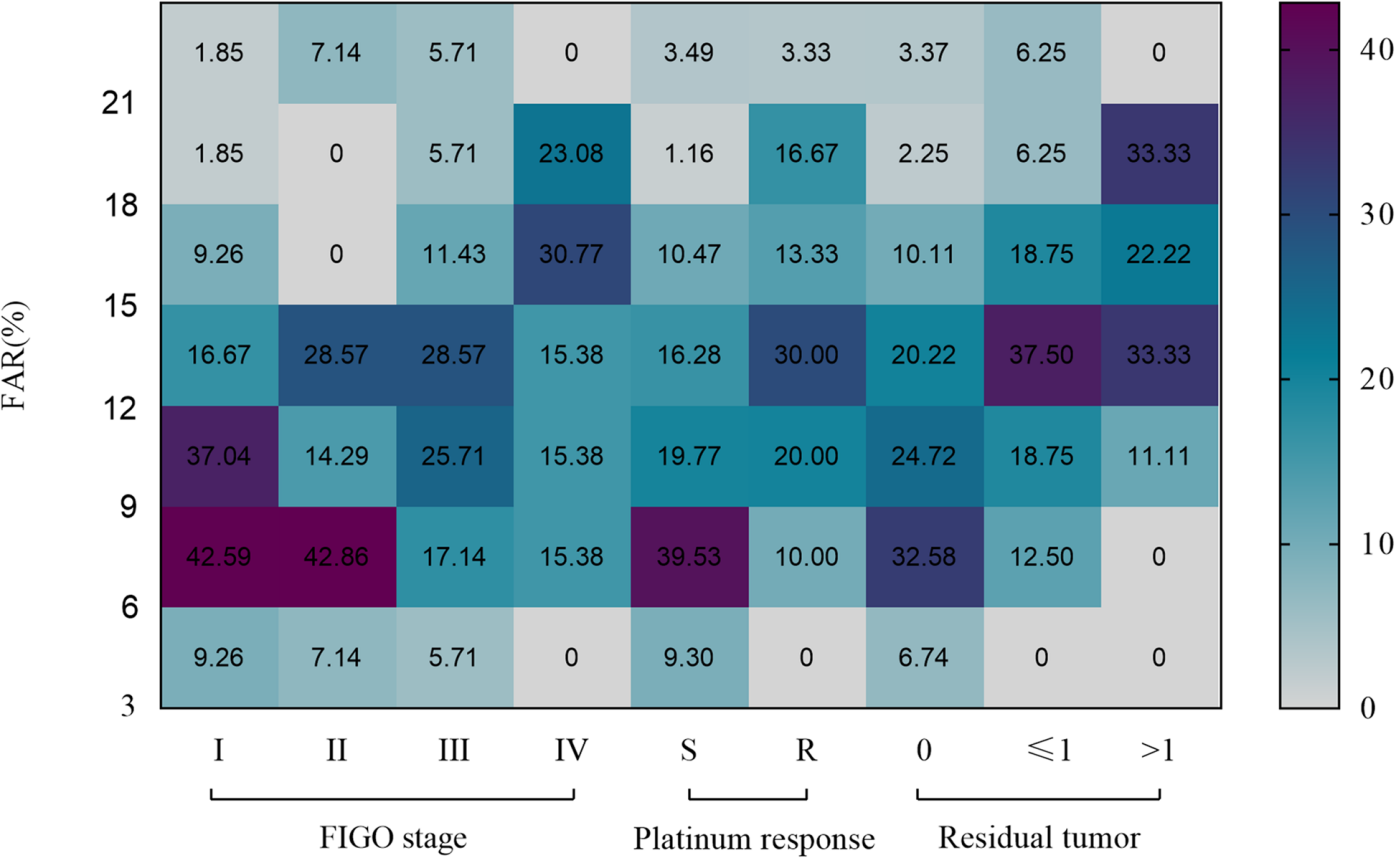
Differential expression of fibrinogen/albumin ratio in patients with different FIGO stages, platinum response, and residual tumour. (Abbreviations: S, platinum-sensitive; R, platinum resistance)

Table 1 shows the median and range of preoperative biochemical variables (CA125, HE4, albumin, fibrinogen, D-dimer) stratified by the clinicopathological features of the 114 patients. The values of these indicators are significantly different in patients with platinum sensitivity and platinum resistance. FAR was associated with endometriosis, stage, residual tumor and platinum response. The distribution and proportion of FAR (%) are shown in Fig. 2.

**Table 1:** Patient characteristics in relation to preoperative blood parameters

Characteristic	N^a^ (%)	CA125^b^ (U/ml)	HE4 ^b^	ALB^b^ (g/l)	FIB ^b^ (g/l)	FAR ^b^ (%)	DDI ^b^
			(pmol/L)				(mg/l)
All cases	114	186.1 (6.5–5000*)	106.2 (37.0–1500*)	41.1 (25.9–52.1)	4.58 (1.94–8.94)	10.7 (4.8–25.1)	1.5 (0.1–55.2)
Age (year)							
< 40	12 (10.5)	254.7 (66.2–912.8)	111.1 (35.5–209.6)	42.2 (33.7–49.2)	4.85 (2.69–6.59)	11.4 (6.5–16.3)	2.1 (0.7–10.7)
40 ~ 59	75 (65.8)	131.0 (6.5–2854.0)	106.2 (37.0–1500*)	41.5 (28.1–52.1)	4.34 (1.94–8.94)	10.2 (4.9–25.1)	1.4 (0.1–55.2)
≥60	27 (23.7)	239.1 (13.29–5000*)	124.3 (55.5–1500*)	40.3 (25.9–46.5)	4.38 (2.42–8.79)	12.1 (6.2–23.3)	2.3 (0.2–30.6)
*P* value		0.081	0.228	0.377	0.778	0.074	0.451
FIGO stage							
I	53 (46.5)	66.2 (6.5–5000*)	106.2 (37.0–1500*)	42.1 (28.1–52.1)	3.90 (1.94–7.15)	8.9 (4.9–21.6)	0.7 (0.1–42.6)
II	13 (11.4)	133.5 (22.2–1401.0)	106.2 (47.0–252.4)	43.7 (31.5–47.8)	4.04 (2.30–7.92)	9.1 (5.2–25.1)	1.3 (0.3–55.2)
III	35 (30.7)	283.8 (46.1–2854.0)	106.2 (47.4–1500*)	40.3 (30.8–46.5)	4.83 (2.20–8.94)	12.0 (5.4–23.3)	3.2 (0.5–11.0)
IV	13 (11.4)	304.8 (48.9–5000*)	106.2 (44.1–331.9)	39.2 (25.9–49.2)	4.75 (3.42–7.95)	15.1 (8.8–11.5)	3.9 (0.6–9.2)
*P* value		**0.006**	0.837	**0.018**	0.180	**0.007**	**0.008**
Residual tumour							
0	89 (78.1)	126.0 (6.5–5000*)	106.2 (37.0–1500*)	41.7 (28.1–52.1)	4.13 (1.94–8.94)	9.9 (4.9–25.1)	1.2 (0.1–55.2)
≤1 cm	16 (14.0)	551.9 (46.1–1845.3)	112.7 (68.9–904.0)	39.0 (29.0–45.4)	4.98 (3.25–8.79)	12.9 (7.3–15.9)	3.9 (0.7–9.3)
> 1 cm	9 (7.9)	475.1 (151.6–1866.0)	209.6 (47.4–1500*)	39.2 (25.9–49.2)	5.46 (3.42–7.95)	15.3 (8.9–20.3)	5.0 (0.8–11.0)
*P* value		**0.034**	0.238	0.056	**0.028**	**< 0.001**	**0.009**
Endometriosis							
Present	33 (28.9)	121.0 (22.2–5000*)	99.5 (44.1–1500*)	41.6 (35.7–52.1)	3.83 (2.20–7.15)	8.4 (5.0–20.0)	1.3 (0.2–42.6)
Absent	81 (70.1)	195.0 (6.5–5000)	106.2 (37.0–1500*)	40.7 (25.9–49.9)	4.72 (1.94–8.94)	11.5 (5.0–25.0)	2.1 (0.1–55.2)
*P* value		0.314	0.111	0.090	**0.022**	**0.010**	0.593
Platinum response							
Sensitive	85 (74.6)	105.5 (6.5–5000*)	106.2 (37.0–1500*)	41.7 (28.1–52.1)	4.00 (1.94–8.94)	9.2 (4.9–25.1)	1.0 (0.1–30.6)
resistant	29 (25.4)	378.2 (69.93–1866.0)	134.3 (47.0–1500*)	39.2 (25.9–49.2)	4.99 (2.71–8.79)	13.3 (6.2–23.3)	5.0 (0.6–55.2)
*P* value		**0.002**	**0.028**	**0.005**	**0.002**	**0.001**	**< 0.001**

*Abbreviations*: *ALB* albumin, *FIB* fibrinogen, *FAR* fibrinogen/ albumin ratio, *DDI* d-dimer

^a^ Categorical data are shown in absolute value and proportion

^b^ Continuous variables are represented by median and range

* The upper limit of CA125 detection is 5000. The upper limit of HE4 detection is 1500

*P* values with statistical significance were denoted

**Fig. 3:**
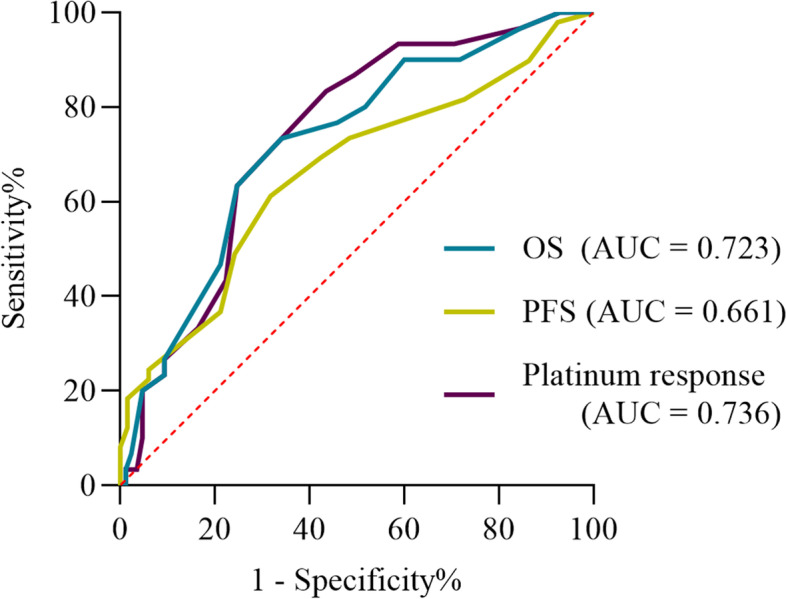
Receiver operating characteristic curve demonstrating the AUC of preoperative FAR for platinum resistance, PFS and OS. (Abbreviations: AUC, area under the curve; FAR, fibrinogen/albumin ratio; OS, overall survival; PFS, progression-free survival)

The ROC curve predicting the outcome of platinum-based chemotherapy was generated, of which the area under the curve (Fig. 3) was 0.736 to verify that the optimal cutoff point FAR was 12%. The sensitivity was 73.3%, and the specificity was 68.2%. Among 114 patients included in the analysis, 63 patients had FAR< 12%, and 51 patients had FAR ≥12%. The relationships between preoperative peripheral blood variables and platinum response are presented in Table 2. On univariate analysis, variables such as FIGO stage (hazard ratio (HR) = 2.526, *P* < 0.001), albumin (HR = 0.903, *P* = 0.055), fibrinogen (HR = 1.516, *P* = 0.016), D-dimer (HR = 1.116, *P* = 0.027), and FAR ≥12% (HR = 5.012, *P* = 0.003) were risk factors for platinum resistance in patients with OCCC. In multivariate analysis, FAR ≥12% (HR = 4.963, *P* = 0.002) and D-dimer were independent risk factors for platinum resistance.

**Table 2:** Univariate and multivariate analysis regarding platinum response. (*N* = 114)

Variables	Univariate analysis			Multivariate analysis		
	HR	95% CI	*P*	HR	95% CI	*P*
stage	2.526	1.503–4.248	**< 0.001**			0.114
Endometriosis			0.365			
CA125			0.703			
HE4			0.700			
ALB	0.903	0.814–1.002	**0.055**			0.567
FIB	1.516	1.079–2.130	**0.016**			0.661
FAR(%)						
< 12	1			1		
≥12	5.012	1.737–14.464	**0.003**	4.963	1.845–13.354	**0.002**
DDI	1.116	1.103–1.23	**0.027**	1.101	1.001–1.210	**0.048**

*Abbreviations*: *ALB* albumin, *FIB* fibrinogen, *FAR* fibrinogen/ albumin ratio, *DDI* d-dimer, *HR* hazard ratio, *CI* confidence interval

*P* values with statistical significance were denoted

### Association between FAR and complete cytoreduction in the advanced stage population

Of the 48 advanced stage patients, 56.3% had an FAR ≥12%. The overall complete resection rate was 47.9, and 60.9% in patients with FAR< 12%. As shown in Table 1, FAR, CA125, D-dimer and FIB were associated with residual tumors. In the advanced-stage population, an FAR≥12% (HR = 4.000, *P* = 0.025) was further validated as an independent prognostic factor for failure to achieve complete resection after multivariate analysis (Table 3).

**Table 3:** Multivariate analysis regarding residual tumour of advanced patients. (*N* = 48)

Variables	Multivariate analysis		
	HR	95% CI	*P*
stage			0.501
Endometriosis			0.124
CA125			0.765
FIB			0.250
FAR (%)			
< 12			
≥12	5.057	1.903–13.440	**0.001**
DDI			0.707

*Abbreviations*: *FIB* fibrinogen, *FAR* fibrinogen/ albumin ratio, *DDI* d-dimer, *HR* hazard ratio, *CI* confidence interval

*P* values with statistical significance were denoted

### Association between FAR and survival in patients with OCCC

In addition to its predictive value for the platinum response, the FAR was also a predictor of recurrence and death within three years. The optimal cutoff point of FAR is still 12%. The ROC curve is also shown in Fig. 3.

**Fig. 4:**
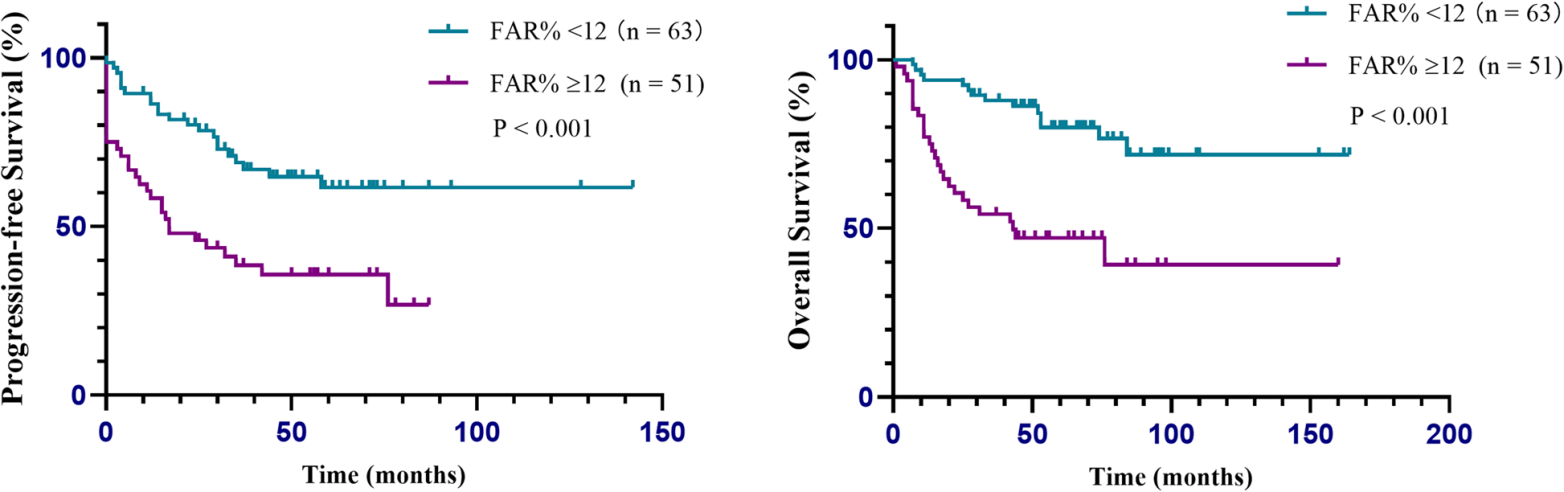
Kaplan-Meier curves of PFS and OS for all patients stratified into two groups: patients with a FAR ≥12% and patients with a FAR < 12%

**Fig. 5:**
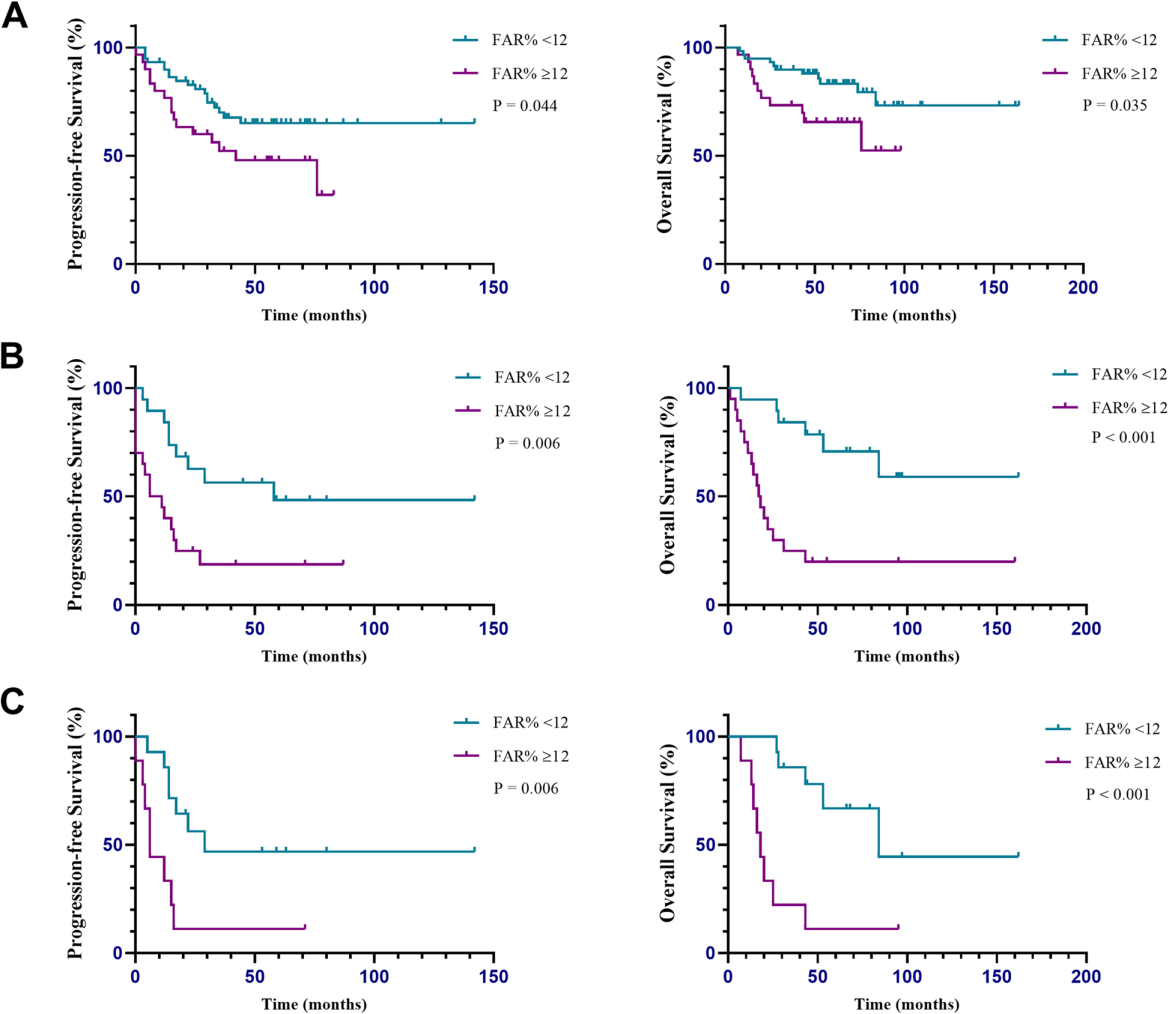
Kaplan–Meier curves of PFS and OS for (**A**) patients without residual tumour, (**B**) patients with advanced-stage and (**C**) advanced patients without residual tumour stratified into two groups: patients with a FAR ≥12% and patients with a FAR < 12%

At the end of the follow-up period (May 30, 2021), 44 patients (41.9%) had recurrence, and 31 patients (29.5%) died. The median follow-up time was 52 months (range, 1–164 months). The relationships between preoperative biochemical variables and survival outcomes are presented in Table 4. A FAR ≥12% was an independent negative factor for both PFS (HR = 2.228, *P* = 0.009) and OS (HR = 3.606, *P* < 0.001), as well as high D-dimer, advanced stage and residual tumor. The median PFS of all patients was 76 months, and the median OS was not achieved. As shown in Fig. 4, the median PFS and OS were 17 and 43 months in the patients with FAR ≥12%, respectively. However, median survival was not achieved in patients with FAR < 12%. Survival was significantly worse for the patients with FAR ≥12% than for those with FAR < 12%. Subgroup analyses were conducted based on patients without residual tumor material, advanced stage (FIGO III-IV) and advanced patients who achieved no residual disease. Consequently, a high FAR (≥ 12%) showed a stronger correlation with poor OS and PFS across all subgroup analyses (Fig. 5A, B, C).

**Table 4:** Multivariate cox proportional analysis regarding overall survival and progression free survival

Variables	PFS			OS		
	HR	95% CI	*P*	HR	95% CI	*P*
FIB			0.902			0.922
DDI	1.045	1.013–1.078	**0.005**	1.044	1.001–1.089	**0.046**
CA125			0.281			0.143
ALB			0.652			0.593
HE4			0.667			0.587
Endometriosis			0.629			0.947
stage						
I	1			1		
II	3.817	1.526–9.551	**0.004**			0.951
III	7.760	3.313–18.173	**< 0.001**	8.586	3.234–22.796	**< 0.001**
IV	3.590	1.251–10.308	**0.018**	3.596	1.123–11.510	**0.031**
Residual tumour						
0	1					
<= 1 cm			0.410			0.669
> 1 cm	5.556	2.079–14.843	**0.001**	2.484	1.020–6.050	**0.045**
FAR						
< 12%	1					
> = 12%	2.228	1.217–4.081	**0.009**	3.606	1.759–7.390	**< 0.001**

*Abbreviations*: *ALB* albumin, *FIB* fibrinogen, *FAR* fibrinogen/ albumin ratio, *DDI* d-dimer, *HR* hazard ratio, *CI* confidence interval

*P* values with statistical significance were denoted

## Discussion

OCCC is known to be less sensitive to platinum-based front-line chemotherapy and have an overall poor prognosis compared to other types of epithelial ovarian cancers, especially in advanced stages [6]. To date, few clinical and biological biomarkers have been used to predict platinum resistance in OCCC. CA125, fibrinogen, albumin and D-dimer are routine indicators for preoperative examination, making these biomarkers inexpensive and clinically practical. In our previous study, we found that elevated D-dimer and decreased albumin are potential biomarkers for a worse response to front-line platinum chemotherapy and poor clinical outcome in IC-IV stage OCCC patients [18]. However, the combination of D-dimer and albumin did not show better predictive potential for prognosis. The current study extended the patients’ enrolment to any stage of OCCC patients who underwent initial surgery. D-dimer was also shown to be significantly associated with prognosis, which is very consistent with findings in other malignancies [11, 19, 20]. D-dimer is the degradation product of fibrin and fibrinogen, and the increase in D-dimer is closely related to fibrinogen. The current study is the first publication evaluating the predictive and prognostic role of the FAR in OCCC. Encouragingly, FAR, as a compound marker of fibrinogen and albumin, has a fairly consistent potential for prognostic evaluation of platinum response and survival outcomes.

In the present study, the patients with an FAR ≥12% had a higher proportion of stage III-IV tumor, platinum resistance and a lower proportion of no residual tumour than the patients with an FAR < 12%. Among advanced ovarian cancers, several studies have reported the best prognosis for patients receiving complete resection [21–23]. Approximately half of OCCC patients were at an advanced stage with widespread intra-abdominal disease in our cohort. FAR showed significant differences between the two groups with or without complete resection. This indicates that OCCC patients with a relatively high FAR may have a high tumor burden and higher tumor invasiveness and may have more difficulty achieving complete resection. An FAR≥12% is expected to be a complement to predictive models of noninvasive methods.

After a median follow-up of 52 months, the patients with an FAR ≥12% exhibited worse survival than the patients with an FAR < 12%. For the same tumor stage or residual tumor, FAR ≥12% was closely associated with poorer OS and PFS. The results suggested that a preoperative elevated FAR was a useful predictor of a higher risk of recurrence and poor survival in patients with OCCC.

Albumin has been considered an indicator that could not only reflect nutritional status but also be involved in the evaluation system related to the inflammatory response [24]. Hypoalbuminemia can cause immune deficiency in tumor patients, reduce the therapeutic effect and increase mortality [25, 26]. Many observations have shown that fibrinogen, fibrin, and components of the fibrinolytic system have a complex interaction with cancer growth and metastasis [10]. In simple terms, plasma hyperfibrinogen promotes the hypercoagulable state, as well as the adhesion and survival of tumor cells, leading to the metastatic potential of cancer [27]. Fibrinogen can provide a stable framework for the tumor extracellular matrix to promote the adhesion and invasion of tumor cells [28], and block the killing function of natural killer cells to increase the tumor metastasis [29]. In addition, increased levels of circulating fibrinogen can induce the synthesis of interleukin 6 (IL-6) and promote tumor progression [30]. It is known that many OCCC occur in the context of endometriosis. There is evidence that IL-6 expression may be induced by endometriosis inflammation, and long-term exposure of ovarian surface epithelial cells to IL-6-rich endometrioma fluid can induce the expression of gene expression patterns typical of OCCC [31]. This may be one of the pathogeneses of OCCC associated with endometriosis. However, no correlation between endometriosis and the survival of OCCC patients was found in our study, which is consistent with our previous study [32]. In any case, FAR, as a composite index of fibrinogen and albumin, not only represents the inflammatory status but also reflects the nutritional status of the body and plays an important role in the biological behavior related to OCCC through direct and indirect pathways.

This study has several limitations. First, our study is a single-center retrospective study with a relatively small sample size, which might lead to selection bias. Second, some confounding factors may not have been eliminated. Third, the median survival time could not be calculated due to insufficient follow-up time. Therefore, the role of the FAR in predicting the clinical response to platinum-based chemotherapy and the survival of OCCC patients needs to be confirmed by a prospective cohort study with a multicenter design and a large sample size.

## Conclusion

We identified the FAR as an independent predictor of platinum response and survival in OCCC patients after primary surgery. The FAR is a potential prognostic indicator of OCCC due to its easy access and low cost.

## Data Availability

The dataset supporting the conclusions of this article is available upon request. Please contact Prof. Shuang Ye (mendy_ye@126.com) and Prof. Huijuan Yang (huijuanyang@hotmail.com).

## Abbreviations

OCCC: Ovarian clear cell carcinoma
EOC: Epithelial ovarian cancer
FAR: Fibrinogen/albumin ratio
PFS: Progression-free survival
OS: Overall survival
ROC: Receiver operating characteristic
HR: Hazard ratio
mPFS: median PFS
mOS: median OS
IL-6: interleukin 6
